# Directional Selection of Microbial Community Reduces Propionate Accumulation in Glycerol and Glucose Anaerobic Bioconversion Under Elevated pCO_2_

**DOI:** 10.3389/fmicb.2021.675763

**Published:** 2021-06-16

**Authors:** Pamela Ceron-Chafla, Yu-ting Chang, Korneel Rabaey, Jules B. van Lier, Ralph E. F. Lindeboom

**Affiliations:** ^1^Sanitary Engineering Section, Department of Water Management, Delft University of Technology, Delft, Netherlands; ^2^Center for Microbial Ecology and Technology, Ghent University, Ghent, Belgium; ^3^Center for Advanced Process Technology for Urban Resource Recovery, Ghent, Belgium

**Keywords:** high-pressure anaerobic digestion, elevated CO_2_ partial pressure, syntrophic propionate oxidation, *Smithella*, adaptive laboratory evolution

## Abstract

Volatile fatty acid accumulation is a sign of digester perturbation. Previous work showed the thermodynamic limitations of hydrogen and CO_2_ in syntrophic propionate oxidation under elevated partial pressure of CO_2_ (pCO_2_). Here we study the effect of directional selection under increasing substrate load as a strategy to restructure the microbial community and induce cross-protection mechanisms to improve glucose and glycerol conversion performance under elevated pCO_2_. After an adaptive laboratory evolution (ALE) process, viable cell density increased and predominant microbial groups were modified: an increase in *Methanosaeta* and syntrophic propionate oxidizing bacteria (SPOB) associated with the *Smithella* genus was found with glycerol as the substrate. A modest increase in SPOB along with a shift in the predominance of *Methanobacterium* toward *Methanosaeta* was observed with glucose as the substrate. The evolved inoculum showed affected diversity within archaeal spp. under 5 bar initial pCO_2_; however, higher CH_4_ yield resulted from enhanced propionate conversion linked to the community shifts and biomass adaptation during the ALE process. Moreover, the evolved inoculum attained increased cell viability with glucose and a marginal decrease with glycerol as the substrate. Results showed differences in terms of carbon flux distribution using the evolved inoculum under elevated pCO_2_: glucose conversion resulted in a higher cell density and viability, whereas glycerol conversion led to higher propionate production whose enabled conversion reflected in increased CH_4_ yield. Our results highlight that limited propionate conversion at elevated pCO_2_ resulted from decreased cell viability and low abundance of syntrophic partners. This limitation can be mitigated by promoting alternative and more resilient SPOB and building up biomass adaptation to environmental conditions via directional selection of microbial community.

## Introduction

Volatile fatty acids (VFA) are important chemical building blocks obtained by mixed culture fermentation in the carboxylate platform (Holtzapple and Granda, 2009; Agler et al., 2011). However, its accumulation is a symptom of reactor malfunctioning in anaerobic digestion (AD), attributed to dissimilarities between acidogenic, acetogenic, and methanogenic bio-conversion rates (Hickey and Switzenbaum, 1991). The identified causes leading to VFA accumulation are associated with substrate overload (Amorim et al., 2018), possible toxicity due to increasing concentrations of undissociated acids at low pH (Russell and Diez-Gonzalez, 1998), and disparities in the proportionality of acidogenic, syntrophic, and methanogenic microorganisms (McMahon et al., 2004; Li et al., 2016; Town and Dumonceaux, 2016). When VFA production is targeted under anaerobic conditions, the product spectrum selectivity is influenced by operational parameters such as temperature, substrate type, concentration, pH, solids retention time (SRT) and headspace composition (Arslan et al., 2016; Zhou et al., 2018; Wainaina et al., 2019).

Possible steering effects of headspace composition in AD could be magnified applying the auto-generative high-pressure AD technology, which was developed for simultaneous biogas production and upgrading (Lindeboom et al., 2011; Lemmer et al., 2015). Here, by applying a closed gastight vessel, the reactor pressure auto-generatively increases and a higher CH_4_ content in the biogas is achieved due to enlarged differences in CO_2_ and CH_4_ solubility at high operational pressure. Lindeboom et al. (2013) observed a decline in the propionate oxidation rate at an increased total pressure with a concomitantly increased partial pressure of CO_2_ (pCO_2_). As part of the explanatory mechanism, an acidification process and reversible toxicity linked to carbamate formation were proposed. Both processes occur due to increased aqueous CO_2_ concentration (H_2_CO_3_^∗^) in the liquid medium resulting from enhanced CO_2_ dissolution (Lindeboom et al., 2016). Recently, we investigated additional effects of elevated pCO_2_ on syntrophic conversions occurring in AD (Ceron-Chafla et al., 2020). The observed limited propionate and butyrate conversion under elevated pCO_2_ were explained by a more comprehensive mechanism that encompasses bioenergetics, kinetic and physiological effects.

The effects of elevated pCO_2_ on the cell viability level can be attributed to the detrimental effect that increased H_2_CO_3_^∗^ concentrations have on cell membrane permeability. Leakage of internal components, structural modifications, and internal acidification are part of the explanatory mechanisms (Wu et al., 2007; Garcia-Gonzalez et al., 2010). In bioreactors with a pressurized headspace, the effects of elevated pCO_2_ differ from inert gases, such as N_2_, which does not severely compromise cell viability (Wu et al., 2007). Considerably higher hydrostatic pressures of N_2_ are required to achieve the same inactivation levels as with elevated pCO_2_ (Aertsen et al., 2009). Safeguarding cell viability is necessary since reduced cell density and increased percentage of permeable cells might result in impaired specific conversion rates, causing decreased productivity.

The extent of detrimental effects of increased dissolved CO_2_ concentrations on cell membranes depends on localized conditions. Some microbial species have acid tolerance mechanisms involving enzymatic systems carrying out neutralization reactions, proton pumps and modifications in the cell membrane (Guan and Liu, 2020). In consequence, they can counteract ramping H^+^ concentrations due to H_2_CO_3_^∗^ intracellular dissociation. Some authors observed during sterilization experiments that a reduction in water activity in the liquid medium decreases CO_2_ dissolution thus diminishing its intracellular diffusion (Kumagai et al., 1997; Chen et al., 2017). In other cases, microorganisms induce the synthesis of compounds acting as compatible solutes, such as glutamate, which help to tackle changes in osmolarity and CO_2_ toxicity (Oger and Jebbar, 2010; Park et al., 2020). Additionally, the presence of fats in the medium affects the porosity and structure of the cell wall or membranes, thus limiting CO_2_ penetration (Lin et al., 1994).

Previous studies have shown that directional selection of microbial community through adaptive laboratory evolution (ALE) processes is highly effective to improve stress tolerance and selectively enhance product formation by activation of downregulated pathways (Portnoy et al., 2011; Dragosits and Mattanovich, 2013). However, in mixed culture fermentations changes in pathway predominance to enhance hydrogen production already have been observed after relatively short adaptive evolution processes (46 days) (Huang et al., 2016). In particular, ALE has shown to trigger the development of features such as acid resistance mechanisms (Kwon et al., 2011), as well as other physiological mechanisms to conserve cell membrane integrity. Such insights could act as cross-protection mechanisms against stressful pCO_2_ levels and improve metabolic activity.

Elevated pCO_2_ has also shown effects at the ecological level in anaerobic communities. In terms of structure and taxonomic diversity, the overall response to high CO_2_ concentrations included a decrease in richness and lowering diversity, dependent on the actual prevailing pH (Gulliver et al., 2014; Fazi et al., 2019). Moreover, there is evidence of shifts in bacterial and archaeal predominant groups since CO_2_ can be incorporated as a reactant to a different extent and consequently, enhances the metabolic pathways in a selective manner (Gulliver et al., 2014; Yu and Chen, 2019). Therefore, the modifying effect of elevated pCO_2_ in highly redundant communities, such as the ones from anaerobic digesters, might redefine the metabolic activity output and overall process performance.

Elevated CO_2_ concentrations could steer specific metabolic pathways in anaerobic conversion systems, particularly those reactions coupled to the phosphoenolpyruvate (PEP)–pyruvate–oxaloacetate (OAA) node in the biochemical conversions. In addition, high pCO_2_ could promote carboxylation reactions, i.e., propionate formation is favored over acetate formation since the latter will require a decarboxylation reaction of acetyl-CoA (Baez et al., 2009). The enhancement of methane production at high CO_2_ levels could occur due to the combination of homoacetogenic activity coupled with aceticlastic methanogenesis when an electron sink is required (Bajón Fernández et al., 2019). Otherwise, under the stoichiometric provision of a suitable electron donor, such as hydrogen or formate, it will directly promote hydrogenotrophic methanogenesis (Zabranska and Pokorna, 2018).

Glucose and glycerol conversion, under anaerobic conditions, share the glycolytic pathway after glycerol has been converted to glyceraldehyde-3-phosphate and subsequently to PEP (Saint-Amans et al., 2001) (Supplementary Figure 1). These substrates differ in their degree of reduction: for glycerol, this value corresponds to –0.67 electron equivalents/C-mol, whereas for glucose this value is 0. Due to this, glycerol fermentation leads to the formation of reduced compounds and less acetate and CO_2_ as in the case of *Propionibacterium acidipropionici* (Zhang et al., 2016). With the formation of more reduced fermentation products, the redox balance is maintained and biomass growth and overall productivity are sustained (Himmi et al., 2000). In terms of biomass yields, the substrates also have contrasting differences: the theoretical values are higher for glucose than for glycerol being 0.239 vs. 0.145 C-mol biomass/C-mol electron donor, respectively (Heijnen and Kleerebezem, 2010). Moreover, the enzymatic activity associated with CO_2_ fixation also differs in cells grown in glycerol due to the expression of pyruvate carboxylase, which has not been observed with glucose (Parizzi et al., 2012).

These fundamental differences between glucose and glycerol fermentation make a comparative study highly relevant to elucidate the potential of elevated pCO_2_ as a steering parameter in anaerobic conversions when performance limitations need to be overcome. Therefore, in this work, we applied a directional selection process based on increasing substrate load to restructure the microbial community and activate cross-protection mechanisms to enhance the anaerobic conversion of glucose and glycerol under elevated pCO_2_. As a negative control, we investigated the effect of elevated pCO_2_ on the original inoculum. We expected differences in the product spectrum as a result of the dissimilar substrate oxidation state and increased pCO_2_ favoring propionate production. Nonetheless, further propionate oxidation (Pr-Ox) would be limited due to thermodynamic constraints on syntrophic Pr-Ox in relation to interspecies hydrogen transfer (Stams et al., 1998). Further constraints will be established due to the negative effects of elevated pCO_2_ on cell viability and relative abundance of methanogens and syntrophic propionate oxidation bacteria (SPOB) in the original inoculum. The evolved inoculum would feature enhanced cell viability, a higher proportion of fermenters and more resilient SPOB and methanogenic groups. These factors could help to circumvent thermodynamic and performance limitations present in the original inoculum, thereby enabling propionate conversion under elevated pCO_2_.

## Materials and Methods

### Inoculum

Flocculent anaerobic sludge from an anaerobic membrane bioreactor (AnMBR) treating wastewater from a chocolate and pet food industry was used as the starting inoculum. Measured physicochemical parameters are presented in Table 1.

**TABLE 1:** Physicochemical characterization of the original anaerobic inoculum used for the experiments of glucose and glycerol conversion under elevated pCO_2_ and evolved inoculum after adaptive laboratory evolution (ALE) using glucose and glycerol (1 g COD L^–1^) at *T* = 35°C, initial pCO_2_ = 0.3 bar and initial pH in the range 7.5–8.0.

**Parameter**	**Unit**	**Original inoculum**	**Glucose-evolved inoculum**	**Glycerol-evolved inoculum**
		**Mean ± SD (*n* = 3)**	**Mean ± SD (*n* = 3)**	**Mean ± SD (*n* = 3)**
TCOD	mg/L	22,200 ± 1000	3,500 ± 550	6,800 ± 650
SCOD	mg/L	1,900 ± 400	678 ± 65	162 ± 4
TOC	mg/L	7,700 ± 800	681 ± 6	705 ± 3
TSS	g/L	15.9 ± 0.5	3.5 ± 0.1	6.0 ± 0.1
VSS	g/L	13.6 ± 0.1	2.9 ± 0.3	4.9 ± 0.1
VSS/TSS	%	86	81	82
NH_4_-N	mg/L	107 ± 2	269 ± 22	281 ± 7
TP	mg/L	112 ± 1	17 ± 2	28 ± 1
pH	–	7.3	7.2	7.2

### Reactor Set-Up and Operation

Batch experiments with original inoculum were conducted at four different pCO_2_, namely 0.3, 3, 5, and 8 bar initial pressures. Schott bottles with a working volume of 250 mL, air-tight sealed with rubber stoppers were used for the experiments at atmospheric conditions, i.e., 0.3 bar pCO_2_. The employed gas to liquid ratio was 2:3. Stainless steel reactors working in a pressure range of 1–600 bar (Nantong Vasia, China) were used for the experiments at moderately elevated pressure, i.e., 3, 5, and 8 bar. These reactors are fitted with gas and liquid sampling ports in the head, as well as a glycerin manometer. The working liquid volume, in this case, was 120 mL, and the same gas to liquid ratio as before was kept. Reactors were inoculated with 2 g VSS L^–1^. The liquid medium added to each reactor contained 1 g glucose or glycerol as COD L^–1^, macronutrients, micronutrients both prepared according to García Rea et al. (2020) and buffer solution (100 mM as HCO_3_^–^). The initial pH of all the reactors was not adjusted and values were in the range 7.5–8.0.

**FIGURE 1:**
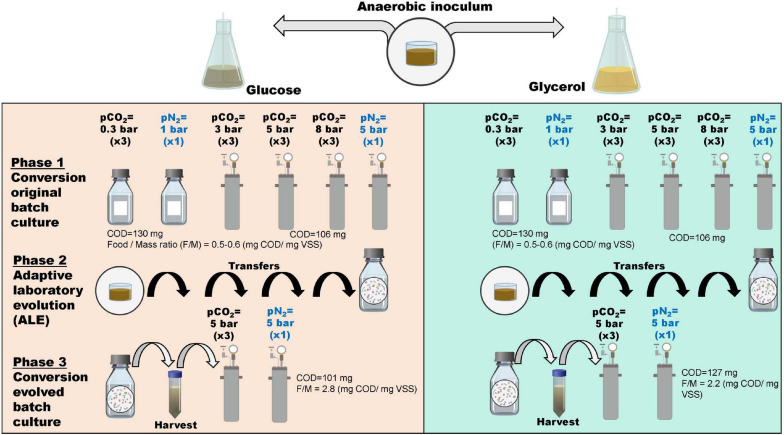
Graphical summary of experimental conditions for the anaerobic conversion experiments at elevated pCO_2_ using glucose and glycerol as substrates. Experiments are organized according to substrate and inoculum conditions.

After filling and closure, atmospheric reactors were flushed for 2 min with 100% N_2_ and sequentially with N_2_:CO_2_, 70:30%. Pressure reactors were initially flushed with the same gas mixture as the atmospheric reactors and afterward, consecutive pressurization-release cycles with >99% CO_2_ were applied to ensure initial headspace composition. Reactors were operated for approximately 10 days, kept at 35°C and continuously shaken at 110 rpm. All the experimental treatments were conducted in triplicates. Pressurized controls with only nitrogen in the headspace were additionally included. To diminish sampling interference during the experiment, we applied the same sampling strategy as previously described (Ceron-Chafla et al., 2020). A graphical description of the experimental design is presented in Figure 1.

### Directional Selection of Microbial Community via Adaptive Laboratory Evolution With Increased Glucose and Glycerol Load

The total length of the atmospheric ALE process was 61 days divided into four cycles with duration as follows: the two first cycles lasted 7 days, which corresponded to complete substrate conversion for initial atmospheric experiments at 0.3 bar pCO_2_. However, due to the limited growth and conversion of intermediates identified after the second cycle, it was decided to perform the third and fourth cycles at 0.3 bar pCO_2_ for 21 and 26 days, respectively, to achieve complete conversion. Schott bottles with a working volume of 2,000 mL were used for the ALE incubation. After every cycle, 240 mL were removed, replaced by fresh medium and the bottles were flushed with N_2_:CO_2_, 70:30%. The substrate concentration in the medium refreshing solution was fixed for all the cycles at 1 g COD L^–1^ glucose or glycerol. It should be noted that owing to the serial dilution procedure, biomass was exposed to a deliberately increased substrate load per cycle. The evolved inoculum was harvested after the fourth cycle, employing low-speed centrifugation and resuspension with macro and micronutrient solution. After this, the obtained biomass was characterized in terms of physicochemical parameters (Table 1) and used to inoculate pressure reactors at 5 bar pCO_2_ and controls at 5 bar pN_2_ to evaluate anaerobic conversion performance of evolved microbial biomass at a higher Food to Mass ratio (F:M ratio) (phase 3; Figure 1). The pressurized experiments with evolved inoculum lasted approximately 10 days.

### Microbial Community Analysis

Biomass samples stored at –80°C from the endpoint of the batch experiments were used to evaluate Microbial community dynamics. After thawing, DNA was extracted according to the instructions included in the DNeasy UltraClean Microbial Kit (Qiagen, Germany). The quality and quantity of the obtained DNA were checked through Qubit^®^ 3.0 DNA detection (Qubit dsDNA HS Assay Kit, Life Technologies, United States). Library construction and sequencing by the Illumina platform were conducted by Novogene (Hong Kong). A summary of the internal protocol is presented in Supplementary Information.

### Analyses

Secondary metabolites in the liquid medium (acetate, propionate, butyrate, and valerate) were measured by gas chromatography (7890A GC; Agilent Technologies, United States) according to the method described by Muñoz Sierra et al. (2020). The gas composition of samples stabilized at atmospheric conditions was determined via gas chromatography (7890A GC; Agilent Technologies, United States) using a thermal conductivity detector operated at 200°C and oven temperature ramping from 40 to 100°C. The system operated with two columns: an HP-PLOT Molesieve GC Column 30 m × 0.53 mm × 25.00 μm and an HP-PLOT U GC Column, 30 m, 0.53 mm, and 20.00 μm (Agilent Technologies, United States). The carrier gas was helium at a constant flow rate of 10 mL min^–1^.

Total cell numbers and live/dead cells were assessed by flow cytometry (BD Accuri^®^ C6, BD Biosciences, Belgium) using Milli-Q as sheath fluid. Before measurement, samples were pre-treated as follows: First, samples were diluted (×500) with 0.22-μm filtered phosphate-buffered-saline (PBS) solution. Diluted samples were sonicated in three cycles of 45 s at 100 W and the amplitude at 50%. Subsequently, samples were diluted (×500) and filtered at 22 μm. Immediately after the pre-treatment, the samples were stained with 5% SYBR^®^ Green I or SYBR^®^ Green I combined with propidium iodide (Invitrogen) and incubated at 37°C for 10 min.

pH, total and soluble COD, TSS, and VSS, ammonium and total phosphorus were measured according to standard methods (American Public Health Association, 2005).

### Statistical Analyses

Statistical analyses were carried out in the R software (R Core Team, 2019). After processing the amplicon sequencing data, a table was generated with relative abundances of the different OTUs and their taxonomic assignment of each sample. Normalization of the samples was carried out based on the flow cytometry data (Props et al., 2017). The R packages vegan (Oksanen et al., 2016) and phyloseq (McMurdie and Holmes, 2013) were used for community analysis. Significant differences (*p* < 0.05) in microbial community composition were identified employing pair-wise Permutational ANOVA (PERMANOVA) with Bonferroni correction using the *adonis* function included in the vegan package.

The order-based Hill’s numbers were used to evaluate the alpha diversity in terms of richness (number of OTUs, H_0_), the exponential of the Shannon diversity index (H_1_) and the Inverse Simpson index (H_2_) (Hill, 1973). Beta diversity was evaluated via the Bray–Curtis distance measure (Bray and Curtis, 1957). Spearman’s correlation analysis was performed using the function *cor.test* ().

## Results

The main results of the glucose and glycerol conversion experiments at elevated pCO_2_ using the evolved and original inoculum are summarized in Table 2. Observations are categorized in terms of cell viability, microbial community, and product spectrum and explained in detail in the following sections.

**TABLE 2:** Summary of the observed effects of the Adaptive Laboratory Evolution (ALE) strategy in the performance of anaerobic conversion of glucose and glycerol under 5 bar pCO_2_.

	**5 bar initial pCO_2_**			
	**Glucose**		**Glycerol**	
	**Original**	**Evolved**	**Original**	**Evolved**
Cell density	Decrease in final total and viable cell density	Increase in final total and viable cell density	Increase in final total and viable cell density	Moderate decrease in final cell density and comparable viable cell density between start and endpoint
Microbial community	Low relative abundance (RA) (<1%) of syntrophic groups. Higher RA of *Methanosaeta* compared to *Methanobacterium*	Increased RA of *Smithella* and *Syntrophobacter* (8%). Slight increase in RA of *Methanosaeta* compared to *Methanobacterium*	Higher RA of *Methanosaeta* compared to *Methanobacterium* than in glucose treatments	Highest RA of *Smithella* and *Syntrophobacter* (18%). RA of *Methanosaeta* and *Methanobacterium* comparable to upper limit in original inoculum treatments
Product spectrum and productivity	Propionate accumulation. Higher acetate and butyrate concentration than under atmospheric conditions Low CH_4_ production	Propionate conversion. Higher CH_4_ production than using original inoculum	Propionate accumulation, despicable butyrate production Lower CH_4_ production compared to glucose	Propionate conversion. Higher CH_4_ production than original inoculum and glucose treatment
				

### Effect of ALE Strategy on Glucose Conversion Under Elevated pCO_2_

#### Cell Viability

##### Original inoculum

**FIGURE 2:**
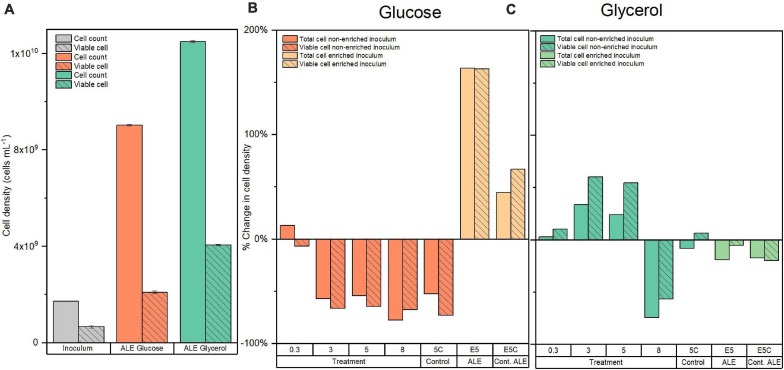
**(A)** Total and viable cell density for the original and evolved inoculum with glucose and glycerol. Percentage of change of total and viable cell density for the **(B)** glucose and **(C)** glycerol conversion experiments at different initial pCO_2_ in the headspace (0.3, 3, 5, and 8 bar) using original inoculum and at 5 bar initial pCO_2_ using evolved inoculum. Pressure controls with N_2_ headspace for the original and evolved inoculum at 5 bar (5C and E5C in the graphs) are included for reference.

The total and viable cell density of the starting inoculum is presented in Figure 2A. During the experiments of glucose conversion at pCO_2_ of 0.3 bar using this inoculum, there was a negligible reduction in the final viable cell density, expressed as percentage change, after 10 days (Figure 2B). However, at moderately high pressures, i.e., 3, 5, and 8 bar initial pCO_2_, the treatments showed a more substantial decrease of approximately 66% in viable cell density for the same period (Figure 2B). It should be noted that nitrogen controls at 5 bar showed a comparable decrease of 73% in viable cell density.

##### Evolved inoculum

Total and viable cell density increased in comparison to the original inoculum after the ALE cycle using glucose as a substrate. In particular, there was a 2.2-fold increase in viable cell density in comparison to the original inoculum (Figure 2A). After the exposure to 5 bar pCO_2_, the viable cell density of the evolved inoculum showed an increase of 163% compared to the original inoculum. Nitrogen controls at 5 bar presented a smaller increase of 67% in viable cell density after 10 days (Figure 2B).

#### Microbial Community

##### Original inoculum

**FIGURE 3:**
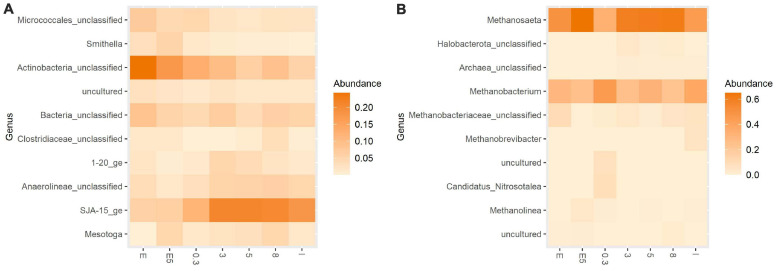
Heatmap presenting the relative abundance of **(A)** Bacterial community and **(B)** Archaeal community at the genus level. Experimental treatments correspond to glucose conversion at elevated pCO_2_ using original (0.3, 3, 5, and 8 bar) and evolved inoculum (5 bar–E5). I corresponds to the original inoculum and E to the evolved inoculum. Pressure corresponds to initial values before equilibrium.

The proportion of bacteria and archaea, based on the total number of processed reads, corresponded to 79 and 21%, respectively, in the original inoculum. The bacterial community of the original inoculum was majorly composed of the phyla Chloroflexi (52%), Actinobacteriota (22%), Firmicutes (10%), and Proteobacteria (5%). In terms of the relative abundance of the bacterial community at the genus level, there were a representative proportion of *SJA-15_ge* (35%) and *unclassified Micrococcales* (19%) (Figure 3A). The proportion of genera associated with syntrophic Pr-Ox namely *Smithella* was low (<1%). The archaeal community was mainly composed of members of the aceticlastic genus *Methanosaeta* (68%) and the hydrogenotrophic genus *Methanobacterium* (31%) (Figure 3B).

In the experiments at 3, 5, and 8 bar initial pCO_2_, bacterial genera from the class Anaerolineae: *SJA-15_ge* (38–45%), *unclassified Micrococcales* (11–16%) were predominant (Figure 3A). The relative abundance of syntrophic groups remained low (<1%). The proportion of total archaea was below 45% in all treatments. Additionally, Figure 3B suggests a contrasting relationship in the relative abundance of *Methanobacterium* and *Methanosaeta* in the treatments when compared to the original inoculum.

##### Evolved inoculum

After the ALE cycles, the proportion of bacteria and archaea was 70 and 30%, respectively. The directional selection of microbial community favored the relative abundance of bacterial phyla Actinobacteria, increasing its abundance to 48% and decreased the proportion of Chloroflexi to 20% of total bacterial reads for this inoculum. At the genus level, the relative abundance of *unclassified Micrococcales* and *SJA-15_ge* corresponded to 44 and 12%, respectively (Figure 3A). *Smithella* increased to 4% of the total bacterial reads. At the archaeal level, the proportions of the two methanogenic genera *Methanosaeta* and *Methanobacterium* corresponded to 68 and 31% of the archaeal reads, respectively (Figure 3B).

For the experiments at 5 bars initial pCO_2_ with evolved inoculum, at the genus level, *unclassified Micrococcales* remained predominant (28%), as well as *SJA-15_ge* (15%) (Figure 3A). Under the imposed experimental conditions, the abundance of syntrophic groups, e.g., *Smithella* (Figure 3A) and *Syntrophobacter*, cumulatively increased to 8% of the total bacterial reads. The proportion of total archaea after exposure to pCO_2_ corresponded to 39% of the total reads. The relative abundance of *Methanosaeta* increased to 77%, whereas *Methanobacterium* remained around 22% of total processed archaeal reads (Figure 3B).

#### Product Spectrum During Glucose Consumption

##### Original inoculum

**FIGURE 4:**
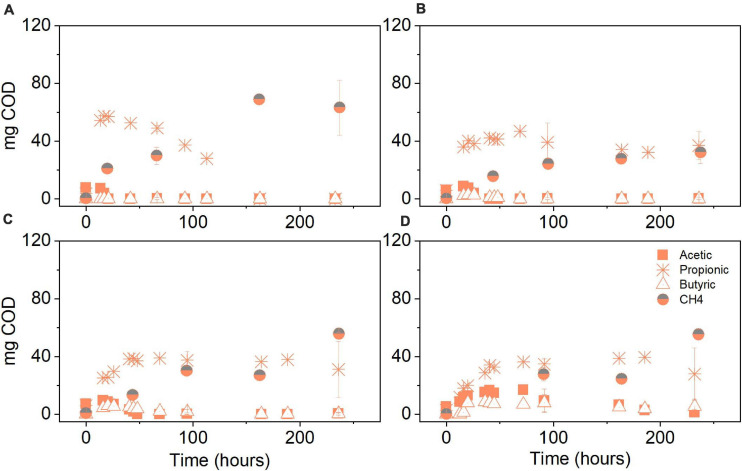
Volatile fatty acid (VFA) and CH_4_ production (mg COD) over time for the glucose conversion experiments using original inoculum at initial pCO_2_ of **(A)** 0.3 bar, **(B)** 3 bar, **(C)** 5 bar, and **(D)** 8 bar. Pressure corresponds to initial values before equilibrium. Data points represent experimental data. Bars represent the standard deviation of three biological replicates measured at the beginning, middle, and end of the experiment.

The product spectrum of the anaerobic conversion of glucose under different initial pCO_2_ levels was mainly composed of propionate, acetate, butyrate and CH_4_. Propionate production was predominant under initial pCO_2_ of 0.3 bar, reaching 399 mg COD–Pr L^–1^, which in terms of the mass balance corresponded to 44% of the initial COD (Figure 4A). In the experiments at 3, 5, and 8 bar pCO_2_, propionate production peaked at approximately 407 ± 32 mg COD–Pr L^–1^, which accounted for 38% of the initial COD. Small discrepancies in the total amount being fed to atmospheric and pressure reactors were experienced since the effective volume differed among reactors to keep the liquid to gas ratio comparable. Acetate and butyrate amounts were higher at initial pCO_2_ of 8 bar compared to atmospheric conditions, whereas at 3 and 5 bar a noteworthy accumulation of these metabolites was not detected (Figures 4B–D). Propionate conversion was hindered by elevated pCO_2_ and in consequence, decreased CH_4_ production was observed in the treatments at high initial pCO_2_. The experiments at 3, 5, and 8 bar pCO_2_ resulted in an average 30% decrease in the final amount of COD–CH_4_ produced in comparison to the atmospheric control at 0.3 bar pCO_2_ evaluated after 10 days (Figures 4A–D).

##### Evolved inoculum

**FIGURE 5:**
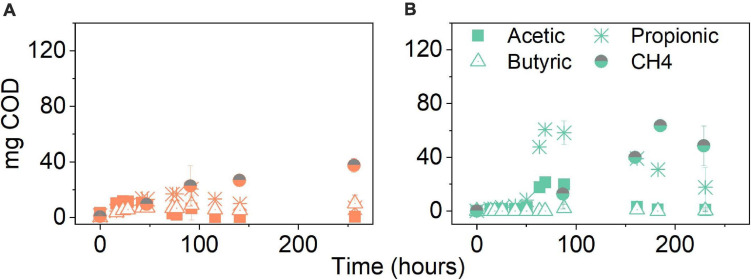
Volatile fatty acid and CH_4_ production (mg COD) over time for **(A)** glucose and **(B)** glycerol conversion experiments at elevated pCO_2_ using evolved inoculum at 5 bar initial pCO_2_. Pressure corresponds to initial values before equilibrium. Data points represent experimental data. Bars represent the standard deviation of three biological replicates measured at the beginning, middle, and end of the experiment.

Figure 5A shows the composition of the product spectrum in the experiments with evolved inoculum which remained similar to the experiments with original inoculum; however, less accumulation of intermediates, particularly propionate, was detected. Propionate production peaked after 92 h (Supplementary Figure 6) and it was no longer detected at significant amounts at the end of the experiment (Supplementary Table 1). A preliminary 33% decrease in the final COD–CH_4_ was calculated when comparing to the treatment at the same pCO_2_, i.e., 5 bar, using the original inoculum (Figure 4C).

### Effect of ALE Strategy on Glycerol Conversion Under Elevated pCO_2_

#### Cell Viability

##### Original inoculum

In the experiments of glycerol conversion at elevated pCO_2_ using the starting inoculum at 0.3 bar pCO_2_, there was a negligible 10% increase in final viable cell density compared to initial conditions. However, at initial pCO_2_ of 3, 5, and 8 bar there was an increase of approximately 50% for the two lowest pressures and a comparable percentage decrease for the highest pCO_2_. Nitrogen controls at 5 bar did not present a considerable change in viable cell density, just accounting for a 6% increase (Figure 2C).

##### Evolved inoculum

After the ALE process with increasing glycerol load, there was a 5.2-fold increase in viable cell density in comparison to the original inoculum and a 0.9-fold increase compared to the ALE with glucose as substrate (Figures 2A,B). After exposure to 5 bar pCO_2_, the evolved inoculum showed a negligible 5% decrease in viable cell density. Nitrogen controls at 5 bar showed a 20% decrease compared to the initial conditions after 10 days (Figure 2C).

#### Microbial Community

##### Original inoculum

**FIGURE 6:**
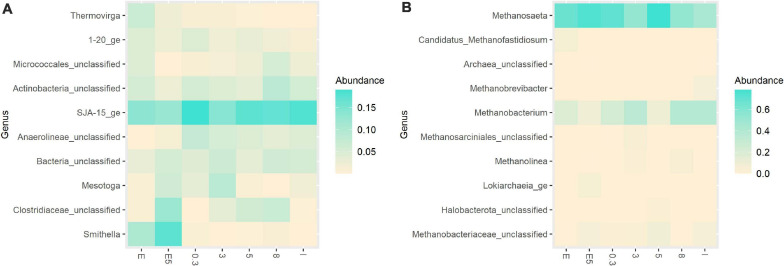
Heatmap presenting the relative abundance of **(A)** Bacterial community and **(B)** Archaeal community at the genus level. Experimental treatments correspond to glycerol conversion at elevated pCO_2_ using original (0.3, 3, 5, and 8 bar) and evolved inoculum (5 bar–E5). I corresponds to the original inoculum and E to the evolved inoculum. Pressure corresponds to initial values before equilibrium.

In the experiments with the original inoculum at 3, 5, and 8 bar initial pCO_2_, results showed a predominance of bacterial genus *SJA-15_ge*, whose relative abundance calculated based on processed reads varied between 34 and 42%, and other genera, such as *Clostridium_sensu_stricto_12* (8–12%), as well as *unclassified Micrococcales* and *Mesotoga* with relative abundances <14% (Figure 6A). The relative abundance of SPOB remained low (<1%) in all cases. There was a descending trend regarding the changes in the proportion of total archaea for the high pCO_2_ experiments using the original inoculum: for experiments at 3, 5, and 8 bar pCO_2_, the archaeal presence corresponded to 35, 22, and 18% of the total number of processed reads, respectively. *Methanosaeta* had the highest relative abundance at the genus level, varying between 58 and 82%, while *Methanobacterium* ranged between 17 and 41% of total processed archaeal reads (Figure 6B).

##### Evolved inoculum

After ALE with glycerol, the proportion of Bacteria and Archaea corresponded to 81 and 19% of processed reads, respectively. The bacterial community composition was dominated by phyla Chloroflexi (35%), Actinobacteriota (19%), Desulfobacterota (14%), and Synergistota (14%). At the genus level, *SJA-15* (27%), *unclassified Micrococcales* (17%), *Smithella* (13%), and *Thermovirga* (11%) showed the highest relative abundances (Figure 6A). The archaeal community was majorly composed of genera *Methanosaeta* and *Methanobacterium* at a corresponding relative abundance of 76 and 22% (Figure 6B).

For the experiments at 5 bar pCO_2_ with evolved inoculum, the relative abundance of *SJA-15_ge* decreased to 19% and *Clostridium_sensu_stricto_12* (21%) was predominant. At this condition, the relative abundance of syntrophic groups, e.g., *Smithella* (Figure 6A) and *Syntrophobacter*, cumulatively increased to 18%. The proportion of total archaea, in this case, showed an increase to 35% of total processed reads. In terms of community composition, it differed from the glucose experiments: a slightly higher proportion of *Methanosaeta* (89%) was observed, whereas *Methanobacterium* represented 10% of processed archaeal reads (Figure 6B).

##### Changes in Microbial Community Structure Due to Exposure to Glycerol and Glucose and Elevated pCO_2_

A basic analysis of alpha diversity via calculation of the Hill numbers showed an overall decrease in the richness (H_0_) of the bacterial community associated with the directional selection process at increasing substrate concentrations, which seemed to be more noticeable in the case of glycerol compared to glucose (Supplementary Figure 2). The inoculum condition (original vs. evolved) only exposed significant differences in terms of the bacterial community structure for the case of richness, H_0_ (*p* = 0.032) when all treatments were analyzed together. In the case of Pielou’s evenness, significant differences were explained by the type of substrate (*p* = 0.044) and not by elevated pCO_2_.

Beta diversity analysis via calculation of the Bray Curtis distance measures revealed significant differences in the community at the highest taxonomic level (Kingdom) because of exposure to elevated pCO_2_ (*p* = 0.01 and *p* = 0.04, respectively). The inoculum condition was significant only to explain the variability of the bacterial community (*p* = 0.07) among all experimental treatments.

#### Product Spectrum

##### Original inoculum

**FIGURE 7:**
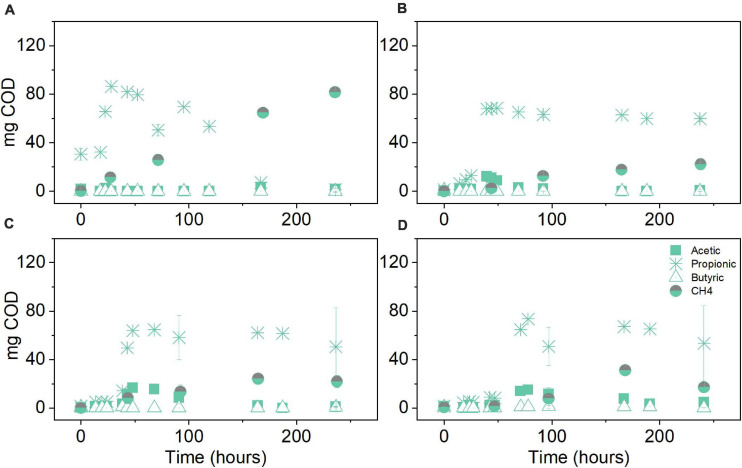
Volatile fatty acid and CH_4_ production (mg COD) over time for the glycerol conversion experiments using original inoculum at initial pCO_2_ of **(A)** 0.3 bar, **(B)** 3 bar, **(C)** 5 bar, and **(D)** 8 bar. Pressure corresponds to initial values before equilibrium. Data points represent experimental data. Bars represent the standard deviation of three biological replicates measured at the beginning, middle and end of the experiment.

The glycerol anaerobic conversion experiments showed a similar final product spectrum to glucose in terms of VFA. The difference was observed in the overall production: propionate in each condition of glycerol fermentation was around two times higher than during glucose fermentation and butyrate production was, on average, four times lower. Under atmospheric conditions, propionate production reached 600 mg COD–Pr L^–1^ at 0.3 bar pCO_2_. In terms of the mass balance, this accounts for 67% of the initial COD (Figure 7A). In the elevated pCO_2_ experiments, the propionate concentration reached its plateau around 647 ± 41 mg COD–Pr L^–1^, corresponding in mass to 57–66% of the initial COD input. Similar to the glucose experiments, propionate conversion was seemingly affected by elevated pCO_2_ leading to its accumulation after approximately 70 h and until the end of the experimental period (Figures 7B–D). Consequently, CH_4_ production was majorly impacted in the glycerol treatments at elevated pCO_2_. On average, methane production was lowered by 69%, compared to the atmospheric control at 0.3 bar pCO_2_. CH_4_ production in the pressurized treatments was, on average, 48% lower in the case of glycerol compared to glucose.

##### Evolved inoculum

In the case of glycerol conversion at 5 bar pCO_2_ using evolved inoculum, propionate peaked after 69 h (Supplementary Figure 6) and decreased until complete conversion was observed by the end of the experiment (Figure 5B). An increment of 55% in final CH_4_ production was achieved when compared to the treatments at the same pCO_2_ using the original inoculum (Figure 7C). Contrary to what was observed with the original inoculum experiments, CH_4_ production from glycerol with evolved inoculum was 23% higher than from glucose at 5 bar pCO_2_.

#### Evaluation of CH_4_ Yield of Glucose and Glycerol Conversion Under Elevated pCO_2_ Using Evolved Inoculum

**FIGURE 8:**
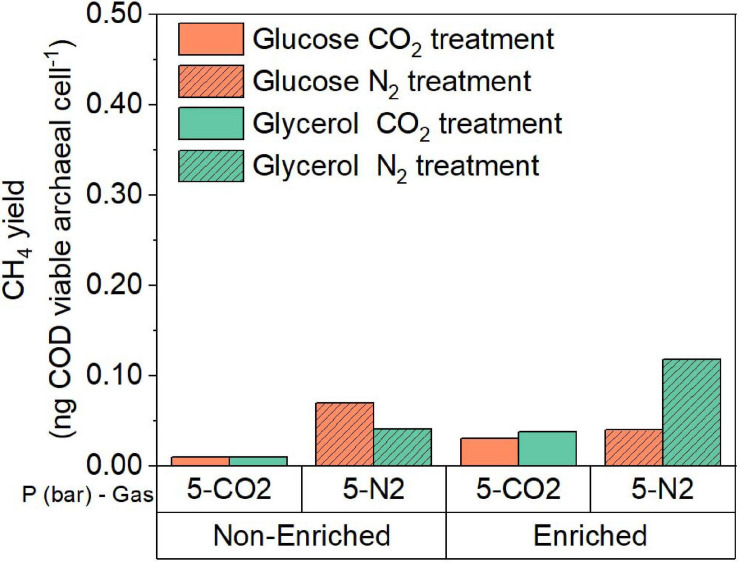
CH_4_ yield normalized by the viable archaeal cell density at the end of the experiment for the glucose and glycerol conversion experiments at elevated pCO_2_ using original inoculum and evolved inoculum at 5 bar. Pressure corresponds to initial values before equilibrium. Nitrogen controls (5 bar) are included as a reference.

The processed sequencing data were used to estimate the proportions of bacteria and archaea in the incubations at atmospheric and pressurized conditions with glucose and glycerol. These proportions together with the results of FCM analysis were used to estimate the theoretical CH_4_ yield in ng of COD per viable archaeal cell for the experimental treatments (Figure 8). Based on these results, we calculated that in the 5 bar pCO_2_ experiment using evolved inoculum, the CH_4_ yield per viable archaeal cell was approximately 2.6 and 4.4 times higher compared to the same condition using the original inoculum for glucose and glycerol, respectively. When comparing glucose and glycerol incubations with evolved inoculum and 5 bar pCO_2_, the CH_4_ yield per cell in the incubation with glycerol was slightly higher than with glucose (1.3 times), which contrasts with the results of the original inoculum. In all cases, elevated pCO_2_ treatments evidenced lower CH_4_ yield than pN_2_ controls. The increased CH_4_ production is likely associated with changes in total archaeal proportion, resulting from the directional microbial community selection process. Furthermore, enhanced product formation is remarkable, since an elevated pCO_2_ of 5 bar seemed to constrain cell growth in the case of the glycerol evolved inoculum (Figure 2C), but not for glucose (Figure 2B). This might suggest the development of different stress-response strategies to elevated pCO_2_ depending on the type of substrate and selected microbial community. Moreover, the relative abundance of *Smithella* + *Syntrophobacter* is 2.3 times higher in glycerol than glucose evolved inoculum (Figures 3A, 6A), which in turn might help to explain higher CH_4_ production due to community and pathway selection despite limited growth. It is postulated that due to the ALE process, a higher F:M ratio employed during the elevated pCO_2_ experiments with evolved inoculum did not constrained bioconversion activity. The F:M ratio was 4–5 times higher in elevated pCO_2_ experiments with evolved inoculum (phase 3, Figure 1) compared to original inoculum (phase 1, Figure 1). However, due to the differences in viable cell concentration, the substrate loads (calculated as mg COD per viable cell), were actually 10 and three times higher compared to the ones using original inoculum for glucose and glycerol, respectively. These differences at the “biomass” and “cell” level could have caused additional inhibition of the biochemical activity of the evolved inoculum, however, results indicate that the ALE process selected for more resilient microorganisms able to cope with the imposed conditions.

## Discussion

From the directional selection of microbial community via ALE, the three main achievements were: (i) an increase in the overall total and viable cell density, (ii) a higher proportion of archaea compared to the original inoculum for glucose and (iii) a larger proportion of SPOB for both substrates (Table 2). These achievements provide a reasonable explanation for enabling propionate conversion and consequently improving CH_4_ production under elevated pCO_2_.

The ALE process could have contributed to the development of protective mechanisms associated with the conservation of cell membrane integrity. It is known that changes in environmental conditions, such as temperature and osmotic pressure, trigger modifications in the structure and fluidity of cell membranes (Beney and Gervais, 2001). Bacteria and archaea differ in the chemical composition of their membrane lipids (Albers and Meyer, 2011), which confers them a distinctive degree of protection toward changes in total pressure and pCO_2_. If membrane fluidity and permeability are being compromised, both groups of microorganisms are capable of adjusting their lipid composition to control ion leakage (Van De Vossenberg et al., 2000). However, previous works suggest that the lipid core of bacterial membranes can adjust itself better to regulate membrane fluidity (Siliakus et al., 2017). This adaptation might confer a survival advantage to bacterial spp. under conditions compromising membrane fluidity and in turn intracellular fluxes, such as the exposure to elevated pCO_2_.

According to Wu et al. (2007), the application of high-pressure CO_2_, i.e., >20 bar, has shown a strong bactericidal effect in cultures of the model organism *E. coli*. The mechanisms contributing to the bactericidal effect included: (i) compromised membrane integrity due to facilitated intracellular diffusion of increased H_2_CO_3_^∗^ concentrations, (ii) drainage of internal cell components such as DNA, and cations as K^+^, Na^+^ that are linked to a more permeable membrane, and (iii) possible internal acidification jeopardizing enzymatic activity as a consequence of a surpassed cytoplasmic buffering capacity (Yao et al., 2014). In our experiments, final pH measurements after decompression (Supplementary Table 1) did not show dramatic differences at the studied pCO_2_ levels because of elevated buffer concentration. It should be realized that the external pH only partly determines cytoplasmic pH, which depends on the physiological features of each microorganism and the internal buffer capacity of the cell. The latter could be compromised by additional neutralization requirements to keep pH homeostasis when H_2_CO_3_^∗^ dissociation occurs in the cytoplasm (Slonczewski et al., 2009). Thus, some degree of cytoplasmic acidification cannot be discarded compromising cell growth in the case of glucose experiments with original inoculum (Figure 2B) and inhibition of microbial activity leading to intermediate propionate accumulation at elevated pCO_2_ for both substrates with the inoculum before the ALE process (Figures 4, 7).

It can be hypothesized that the increased load of an acidifying substrate, such as glucose, in every ALE cycle will lead to the development of protective measures against accumulating acidity, which could help to minimize effects on cell membrane integrity by elevated pCO_2_ (Sun et al., 2005) and could help to explain the increase in cell viability after ALE with glucose (Figure 2B). The noticeable positive effects of using glycerol as the substrate on cell viability (Figure 2C) could be attributed to several factors. Firstly, changes in the medium viscosity can affect the diffusion rate of CO_2_ and could lead to lower microbial inactivation rates by high-pressure CO_2_. A similar observation previously has been reported for a growth medium with increased fat content (Lin et al., 1994). Secondly, researchers recently described that compatible solutes can also act as piezolytes to increase tolerance to pressure exposure (Martin et al., 2002; Scoma and Boon, 2016). Since glycerol can also act as a compatible solute, it might as well confer a temporary piezotolerance depending on its specific uptake and conversion rate. Thirdly, because of its non-ionic nature, glycerol may reduce water activity (a_w_) in the medium, which in turn could contribute to lower microbial inactivation rates by high-pressure CO_2_ as a result of decreased H_2_CO_3_^∗^ formation and a stabilization effect on membrane proteins (Wu et al., 2007; Kish et al., 2012). Regarding the effects of substrate concentration on the a_w_ in the experimental treatments, theoretical calculations performed in the hydrogeochemical software Phreeqc^®^ indeed showed that glycerol lowered the water activity compared to glucose but at elevated substrate concentrations (Supplementary Table 3). At the applied low substrate concentrations of 5 and 9 mM for glycerol and glucose, respectively, it is questionable whether changes in a_w_ would have significantly impacted cell viability in our experiments. Nonetheless, the observed differences in cell viability between glycerol and glucose treatments with the original inoculum (Figure 2), where a higher biomass concentration was applied, might suggest the occurrence of CO_2_ diffusion limitation associated with the presence of glycerol in the medium.

The detrimental effects of high pCO_2_ cannot be solely attributed to a pressure effect. A comparable loss of viability as the one observed at elevated pCO_2_ has up till now only been achieved at hydrostatic pressures higher than 100 MPa (Pagán and Mackey, 2000). When using a non-reactive gas such as N_2_, strong biocidal effects have not been observed even if the pH is significantly lowered to emulate pH levels due to CO_2_ dissolution (Wu et al., 2007). However, we observed a detrimental effect of pressurized N_2_ on the cell viability of evolved inoculum with glycerol (Figure 2C) which suggests a negative effect of headspace pressure at low biomass concentration. Moreover, an increased amount of non-viable cells following pressurized conditions can also be explained by reactor depressurization. It has been shown that pressure release, even if performed gradually, can increase the amount of permeabilized cells (Park and Clark, 2002), and thus, compromising their viability. Further investigations are needed to thoroughly quantify possible decompression effects on cell viability and metabolic activity when using reactive and inert gases such as CO_2_ and N_2_.

At high propionate concentrations and low pH, a microbial community shift toward increased proportions of hydrogenotrophic methanogens has been evidenced, which contributes to maintaining a low partial pressure of hydrogen (pH_2_) to enable Pr-Ox under syntrophic conditions (Li et al., 2018; Han et al., 2020). Apparently, low pH_2_ conditions favor the production of H_2_ instead of NADH from the oxidation of reduced ferredoxin (Fd_red_) (Lee et al., 2008). Furthermore, at low pH_2_, hydrogenotrophic methanogenesis has been described as kinetically (Liu et al., 2016) and thermodynamically more feasible than homoacetogenesis at increasing pCO_2_ (Supplementary Figure 4). This would imply that, in principle, we should have observed an increased proportion of hydrogenotrophic methanogens in the glucose experiments. However, this occurred only in the treatment with the original inoculum at the lowest pCO_2_ of 0.3 bar (Figure 3B). Zhang et al. (2011) described a possible detrimental effect of elevated CO_2_ concentration on the transcription levels of functional [FeFe] hydrogenases of the moderate thermophile *Thermoanaerobacterium thermosaccharolyticum W16*. The reduction in the ratio mRNA expression to 16S DNA gene depended on the type of substrate employed, glucose or xylose, and varied between 66 and 98% (Zhang et al., 2011). Considering this as a plausible hypothesis, H_2_ production might have been hindered in our elevated pCO_2_ experiments, leaving the production of reduced compounds as the main route for NADH consumption.

The biochemical pathways of anaerobic conversion of glucose and glycerol share PEP and pyruvate as central intermediates (Zhang et al., 2015). Pathway steering toward particular electron sinks such as propionate will depend on the environmental conditions and type of microorganism (Supplementary Figure 1). Under the assumption of CO_2_ fixation, the carboxylation from PEP or pyruvate to OAA, which is subsequently further reduced to fumarate, is favored at the reductive branch of the PEP-pyruvate-OAA node (Sauer and Eikmanns, 2005; Stams and Plugge, 2009). Without the presence of sufficient reducing equivalents, a presumed CO_2_ fixation will have a more limited impact on the production of more reduced compounds during glucose fermentation. It should be realized that, compared to glucose, the metabolism of glycerol requires balancing double the amount of reducing equivalents per mole of pyruvate or PEP produced. Under limited hydrogen production, NADH will act as electron carrier, channeling the reducing equivalents toward products such as propionate, whose formation stoichiometrically consumes the NADH from glycerol conversion (Zhang et al., 2015). Concomitantly, acetate production from the acetyl-CoA pathway is downregulated to limit reductive stress due to presence of excess NADH (Doi and Ikegami, 2014). Propionate production from pyruvate in glucose metabolism requires an additional electron donor or extra NADH input. Thus, the simultaneous production of a more oxidized compound, namely acetate, helps to satisfy the redox balance. However, this will occur at the expense of a decreased propionate yield, due to diverged carbon flux (Zhang et al., 2015), helping to explain the differences in propionate levels between the used substrates (Figures 4, 5, 7). Increased propionate production could also be attributed to enhanced enzymatic activities as a result of the substrate choice for the ALE cycles. The activity of pyruvate carboxylase and succinyl CoA: propionyl CoA transferase, both enzymes with a significant role in propionate production, has been enhanced in cultures of *Propionibacterium acidipropionici* using glycerol as the substrate (Zhang et al., 2016). Conversely, this was not observed by these authors when the culture was grown using glucose as the sole carbon and energy source.

The premise of compromised hydrogenase activity because of elevated CO_2_ concentrations could help to explain the decreased proportions of hydrogenotrophic methanogens, particularly in the treatments with glucose. Moreover, the initially high proportion of *Methanosaeta* in the original inoculum (Figure 6B), the reduced H_2_ production when using glycerol as the substrate according to stoichiometry, and a plausible detrimental effect of pCO_2_ on hydrogenase activity, support the idea of the enhancement of a Pr-Ox pathway where thermodynamic limitations associated with interspecies H_2_ transfer plays a less significant role, i.e., the dismutation pathway of *Smithella*, whose relative abundance considerably increased following the ALE process (Figures 3A, 6A). Members of the genus *Smithella* are metabolically active in a broader range of propionate concentrations (Ariesyady et al., 2007), low HRT (Ban et al., 2015) and acidic pH (Li et al., 2018) than members of the genus *Syntrophobacter*. The conditions imposed during the directional selection, i.e., serial transfers to fresh medium, exposed the microorganisms to increased substrate loadings per cell. This possibly contributed to the enrichment of this genus, particularly in the glycerol experiments at 5 bar pCO_2_ using evolved inoculum (Figure 6A). Additionally, if thermodynamic feasibility is considered, the dismutation pathway, i.e., propionate conversion to butyrate and acetate, is less sensitive to the effects of increasing pH_2_ and pCO_2_ than the methyl malonyl-CoA pathway, where propionate is converted to acetate and H_2_, which undergo further conversion by methanogenic archaea (Dolfing, 2013) (Supplementary Figure 5). For further reference, we have summarized the stoichiometries of possible metabolic pathways for the anaerobic conversion of glucose and glycerol including either the dismutation or the methyl malonyl-CoA pathways (Supplementary Table 2).

It is postulated that the occurrence of the *Smithella* pathway in the incubation with both substrates relates well with enhanced CH_4_ yield and might ameliorate end-product inhibition due to elevated pCO_2_ in the case of glucose (Supplementary Table 2). Moreover, less CO_2_ is being produced in the glycerol treatments either by methyl malonyl-CoA pathway or by the dismutation pathway from *Smithella* (Supplementary Table 2). Theoretically, this could enable a substantial CO_2_ fixation via the OAA route (Supplementary Figure 1) and enhanced propionate production if enough reducing equivalents are available. Regulation of the redox balance could be achieved in this pathway by the consumption of reducing equivalents in the intermediate steps, forming malate and succinate. Due to these two reasons, moderately high pCO_2_ levels have likely affected glycerol conversion to a lesser extent.

The here presented higher CH_4_ yields at 5 bar pCO_2_ using evolved inoculum have to be interpreted with caution since significant differences were found in the initial viable cell density of treatments with evolved and original inoculum (*p* = 0.004), as a consequence of the higher microbial F:M ratio imposed to keep the experimental liquid volume comparable in all treatments. The observed lower initial cell density in the experiments with evolved inoculum is attributed to the followed harvesting and resuspension procedure for biomass recovery. These initial differences did not necessarily lead to statistically significant higher proportions of viable biomass (*p* = 0.43) at the end of the experiments using original inoculum, thus comparisons in terms of active biomass are fairly reasonable. The CH_4_-yield calculated per viable cell showed that the methanogenic activity under elevated pCO_2_ was moderately enhanced due to the directional selection process and might be indicative of microbial community resilience to elevated pCO_2_. It was not possible to extrapolate these yields in terms of VSS concentration since this parameter only showed a moderate positive correlation with the log-transformed viable cell density data (*r*_s_ = 0.65, *p* = 0.005) (Supplementary Figure 3) and did not constitute a good proxy for microbial biomass in the experiments here presented. We would not have been able to evidence subtle changes in total cell density and cell viability compromising overall metabolic activity by only relying on this measurement, since it includes dead/non-viable cells and extracellular compounds besides the active biomass (Foladori et al., 2010).

At the microbial ecology level, high CO_2_ concentrations shift the community structure and reduce the taxonomic diversity in different environmental systems (Yu and Chen, 2019), with effects beyond acidification (Gulliver et al., 2014). In our experiments, the directional selection process, prior exposure to elevated pCO_2_, proved to be preponderant for changes in community structure (Hill number H_0_–Richness Supplementary Figure 2A) and overall diversity (Figures 3, 6), which can benefit reactor start-up. Changes in alpha diversity could be linked to a community reorganization as a consequence of the selection pressure (increased substrate load) or an applied disturbance, namely the elevated pCO_2_ (Werner et al., 2011). In our experiments, as expected, microbial community richness decreased due to the ALE process, but VFA production was not compromised. Fermentative activity tends to be conserved even if decreased richness is observed, most likely due to the resilience of this process to stress conditions (Mota et al., 2017). However, conservation of functionality features of the evolved community and its prevalence as the core microbiome in long-term AD-operation will depend on process operation and control (Tonanzi et al., 2018). Concerning the archaeal community structure, CO_2_ enrichment in anaerobic digesters at atmospheric conditions can modify the ratio aceticlastic: hydrogenotrophic methanogens favoring the abundance of *Methanosaeta* (Bajón Fernández et al., 2019). This finding is in alignment with the observations here described and the study by Lindeboom et al. (2016), where a high relative abundance of *Methanosaeta* is reported at increased pCO_2_ levels during high-pressure AD.

Our observations associated with changes in structure and diversity are only indicative of the effects of elevated CO_2_ in fermentative and methanogenic communities and their syntrophic interactions at the bioreactor level, because of the short duration of our experiments. Further support for these conclusions needs to come from longer incubations under pressurized CO_2_ conditions with different types of inocula. Longer incubations leading to increased growth of adapted biomass could enable a more accurate quantification with standard methods (VSS). If a more thorough characterization of the biomass at cell level in terms of average cell dimension and biovolume would be performed, it could permit a better correlation between VSS and FCM results. Similar to previous work, a correlation between protein measurements following the Lowry method and VSS could also provide additional characterization (Lindeboom et al., 2018). Moreover, if these incubations are monitored with online pH, pressure measurements and intensive sampling for microbial community dynamics, a clearer correlation could be obtained between changes in the community and operational variables modified by the pressurized operation. But, even then, the accounted effects might depend on specific system characteristics and operational strategy. Observations of natural soil communities exposed to elevated atmospheric pCO_2_ show that there is not a common agreement over the effects on the microbial ecology. Recent studies have reported either no significant changes (Bruce et al., 2000; Ahrendt et al., 2014) or major shifts in the microbial community (Xu et al., 2013; Šibanc et al., 2014). These shifts include, for example, the predominance of acid-resistant groups such as Chloroflexi and Firmicutes or acetogenic spore-forming Clostridia (Conrad, 2020), which agrees with the results here reported (Figures 3, 6). From these investigations, it can be deduced that other environmental factors such as temperature, pH, nutrient availability as well as CO_2_ final concentration and exposure time will determine the overall fate of the microbial community after exposure to elevated pCO_2_.

## Conclusion

A microbial community directional selection strategy was employed in this study to overcome limited syntrophic Pr-Ox in glucose and glycerol anaerobic conversions under elevated pCO_2_. The pressurized incubations using evolved inoculum showed a dissimilarly enhanced final cell viability, with a stronger positive effect of the ALE in cell viability of glucose incubations. Our results suggest that directional selection of microbial community with increased substrate load per cell triggered mechanisms to preserve cell viability. Moreover, it increased the proportions and enhanced the metabolic activity of microorganisms resilient to increasing propionate concentrations such as SPOB and the interrelated methanogenic community. The increased abundance of the genus *Smithella* accompanied by higher proportions of *Methanosaeta* after incubation with glycerol proved to be beneficial for propionate conversion at elevated pCO_2_. The highest CH_4_ yield per cell at conditions elevated pCO_2_ was observed in the glycerol experiments, which was attributed to enhanced propionate formation, as a way to incorporate CO_2_ and maintain the redox balance via metabolic regulation. Overall, using an evolved inoculum with higher viable cell density and restructured community with a predominance of key microbial groups proved to be a right course of action into surmounting reduced metabolic performance associated with elevated pCO_2_.

## Data Availability Statement

The raw fastq files used to create the OTU table for the microbial community analysis, have been deposited in the National Center for Biotechnology Information (NCBI) database (Accession number PRJNA704575).

## Author Contributions

PC-C designed the experiment, supervised the experiment execution, and wrote the manuscript in its majority. Y-tC performed the experiments, analytical measurements, and contributed to the manuscript writing and editing process. PC-C and Y-tC analyzed the data. JL provided feedback to the experimental design and critically revised the final version of the manuscript. KR constructively revised the final version of the manuscript. RL helped to design, supervised the development of the experiment, and provided constructive feedback to the manuscript. All authors contributed to the article and approved the submitted version.

## Conflict of Interest

The authors declare that the research was conducted in the absence of any commercial or financial relationships that could be construed as a potential conflict of interest.
