# Supplementary material for: Directional Selection of Microbial Community Reduces Propionate Accumulation in Glycerol and Glucose Anaerobic Bioconversion Under Elevated pCO2
Source: Front Microbiol. 2021 Jun 16;12:675763. doi: 10.3389/fmicb.2021.675763 (PMC8242345; doi:10.3389/fmicb.2021.675763)
Supplement: Supplementary file 1 [file Table_1.docx]

**Directional selection of microbial community reduces propionate accumulation in glycerol and glucose anaerobic bioconversion under elevated pCO_2_**

**Pamela Ceron-Chafla^1*^, Yu-ting Chang^1^, Korneel Rabaey^2,3^, Jules B. van Lier^1^, Ralph E.F. Lindeboom^1^**

^1^ Sanitary Engineering section, Department of Water Management, Delft University of Technology, Stevinweg 1, 2628 CN, Delft, The Netherlands.

^2^ Center for Microbial Ecology and Technology (CMET), Ghent University, Coupure Links 653, B-9000 Ghent, Belgium

^3^ Center for Advanced Process Technology for Urban Resource Recovery (CAPTURE), Coupure Links 653, B-9000 Ghent, Belgium

*** Correspondence:**Pamela Ceron-Chafla
p.s.ceronchafla@tudelft.nl

**Keywords:** **High-pressure anaerobic digestion, Elevated CO_2_ partial pressure, Syntrophic propionate oxidation, *Smithella*, Adaptive laboratory evolution.**

Number of tables: 3

Number of figures: 6

Number of pages: 12

Supplementary Material

# Supplementary Data

## Internal protocol for Illumina sequencing Novogene

During the amplification, DNA concentration and purity were first monitored on 1% agarose gels and diluted to 1ng/μL by sterile water. Then, 16S rRNA genes of distinct regions (16SV3-V4) were amplified with the specific primer (e.g. 16S V4: 515F-806R). The chosen PCR products, between 400 to 450 bp, were mixed in equidensity ratios. Then, the mixture PCR products were purified with Qiagen Gel Extraction Kit (Qiagen, Germany). The libraries of the samples, generated with NEBNext® UltraTM DNA Library Prep Kit for Illumina and quantified via Qubit and Q-PCR, were analyzed by the Illumina platform.

Paired-end reads were assigned to samples based on their unique barcode and truncated by cutting off the barcode and primer sequence. Paired-end reads were merged by FLASH (Magoc et al., 2011), and quality filtering on the raw tags was performed under specific filtering conditions to obtain the high-quality clean tags (Bokulich et al., 2013) with the Qiime quality-controlled process (Caporaso et al., 2010). The effective tags were obtained after comparison with UCHIME algorithm (Edgar et al., 2011) the reference database, to detect chimera sequences and subsequent removal of those.

Sequences analysis was performed by Uparse software (Edgar, 2013), using all the effective tags. Sequences with ≥97% similarity were assigned to the same operational taxonomic units (OTUs). The representative sequence for each OTU was screened for further annotation. For each representative sequence, Mothur software was performed against the SSUrRNA database of SILVA Database (Wang et al., 2007) for species annotation at each taxonomic rank (Threshold:0.8~1) (Quast et al., 2012) (kingdom, phylum, class, order, family, genus, species). To get the phylogenetic relationship of all OTUs representative sequences, the MUSCLE algorithm (Edgar, 2004) was applied to compare multiple sequences. OTUs abundance information was normalized using a standard of sequence number corresponding to the sample with the least sequences.

# Supplementary Figures and Tables


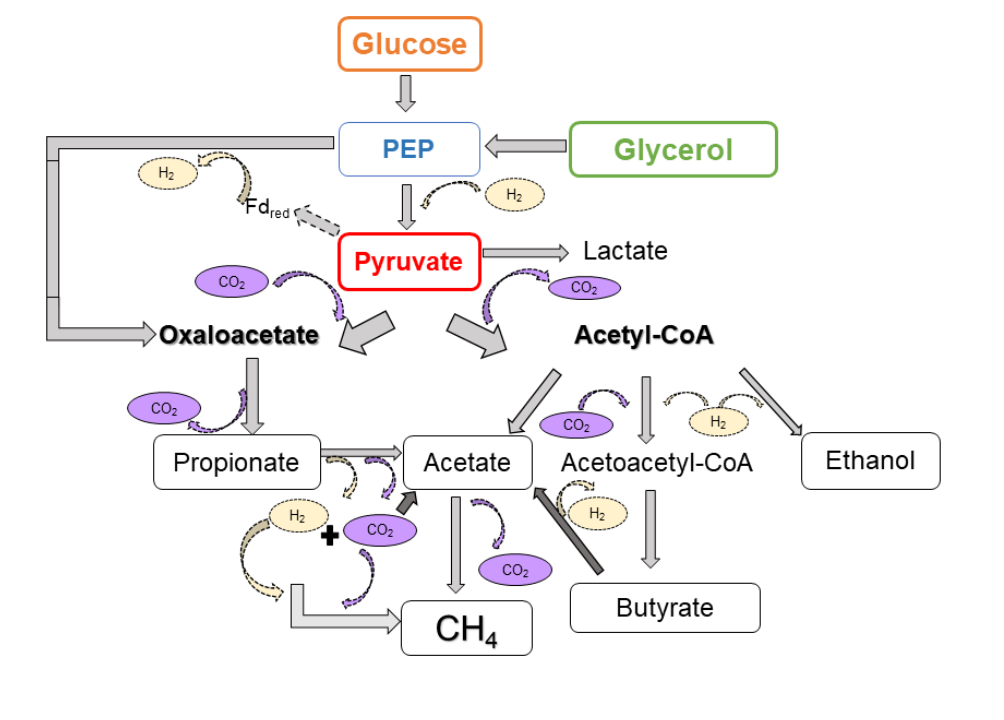


Supplementary Figure 1: Simplified pathway representation for the anaerobic conversion of glucose and glycerol in undefined mixed cultures. As a generalization, reducing equivalents are represented as H_2_. PEP- Phosphoenolpyruvate. Figure adapted from (Zhu et al., 2009; Agler et al., 2011; Ammar et al., 2014).


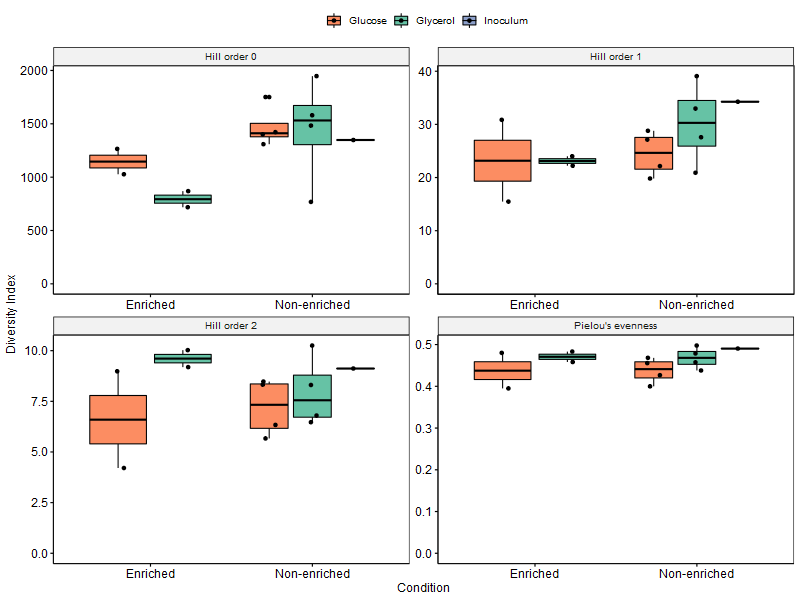


C

D

B

A

Supplementary Figure 2: Boxplots of the alpha diversity index (A) Richness - H_0_, (B) Exponential of the Shannon diversity index - H_1_, (C) Inverse Simpson index - H_2_ and (D) Pielou’s evenness calculated for the experiments of glucose and glycerol anaerobic conversion under elevated pCO_2_ using original and evolved inoculum.


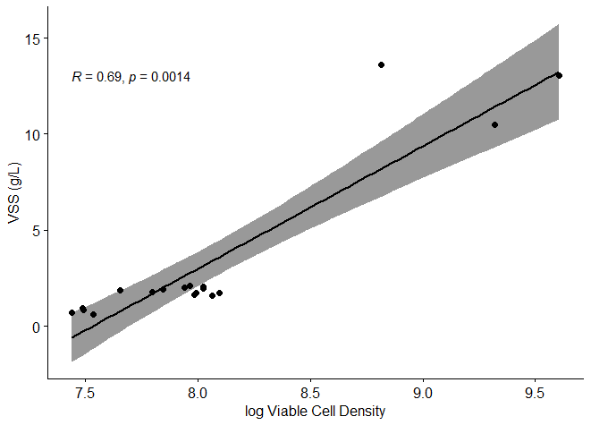


Supplementary Figure 3: Scatter plot showing the correlation between the log-transformed viable cell density and the VSS concentration measured at the end of each experimental treatment and for the original inoculum and the evolved inoculum with glucose and glycerol.





Supplementary Figure 4: Effect of increasing the partial pressure of carbon dioxide in the thermodynamic feasibility of homoacetogenesis and hydrogenotrophic methanogenesis under conditions of low and high partial pressure of hydrogen (pH_2_).





Supplementary Figure 5: Effect of increasing the partial pressure of carbon dioxide (pCO2) (A) and the partial pressure of hydrogen (pH_2_) in the thermodynamic feasibility of propionate oxidation via the methyl malonyl Co-A pathway (C3-oxidation in green) and the *Smithella* (dismutation) pathway (in orange)





**Supplementary Figure 6:** Measured acetate and propionate concentrations (mg/L) during the experiments with the evolved inoculum at 5 bar pCO2 using glucose and glycerol as substrates

Supplementary Table 1: COD balance calculated for each experimental condition for glucose and glycerol conversion under elevated pCO_2_ with original anaerobic inoculum and evolved inoculum. pH value measured after reactor depressurization is additionally provided.

| Substrate | pCO_2_/  pN_2_ (bar) | Acetate  (mg COD) | Propionate  (mg  COD) | Butyrate (mg COD) | Others (mg COD) | CH_4_  (mg COD) | Biomass (mg COD) | Total (mg COD) | Initial substrate (mg COD) | pH |
| --- | --- | --- | --- | --- | --- | --- | --- | --- | --- | --- |
| Glucose | 0.3  (n=3) | ND | ND | ND | ND | 63.2±19 | 33.2 | 96.4 | 130 | 7.2 |
|  | 3  (n=3) | 0.3±0.1 | 36.9±10 | ND | 1.06 | 32.2±8 | 62.4 | 132.9 | 106 | 6.9 |
|  | 5  (n=3) | ND | 40.6±5 | 0.43±0.1 | 2.37±0.6 | 55.8±1 | 81.2 | 180.3 | 106 | 6.6 |
|  | 8  (n=3) | 1.9±0.2 | 36.9±3 | 7.24±4 | 4.07±0.1 | 55.3±4 | 47.1 | 152.4 | 106 | 6.6 |
|  | 5CN*  (n=1) | 1.9 | 2.44 | ND | 0.67 | 120 | 59.8 | 184.6 | 106 | 6.7 |
| Glycerol | 0.3  (n=3) | 0.4±0.1 | ND | ND | ND | 81.6±2 | 66.1 | 148.1 | 130 | 7.2 |
|  | 3  (n=3) | 0.4±0.1 | 59.8±2 | ND | 2.1±0.2 | 22.3±1 | 26.9 | 111.5 | 106 | 6.6 |
|  | 5  (n=3) | 0.7±0.3 | 66.5±6 | ND | 2.3±0.3 | 21.8±5 | 27.6 | 118.8 | 106 | 6.5 |
|  | 8  (n=3) | 5.9±0.5 | 68.8±4 | 1.6±0.8 | 2.9±0.1 | 31.1 | 31.1 | 127.4 | 106 | 6.6 |
|  | 5CN  (n=1) | 1.4 | 2.5 | ND | ND | 94.9 | 15.4 | 114.2 | 106 | 7.6 |
| Glucose (ALE**) | E5  (n=3) | 0.4±0.1 | 7.0±0.8 | 9.9±0.7 | 3.1±0.5 | 37.5±5 | 20.0 | 77.9 | 101 | 6.4 |
|  | E5CN  (n=1) | ND | ND | ND | ND | 51.6 | 6.4 | 58.1 | 101 | 7.5 |
| Glycerol  (ALE) | E5  (n=3) | 0.7±0.1 | 17.6±1.5 | 0.3±0.1 | 0.8±0.1 | 48.5±15 | 26.7 | 94.6 | 127 | 6.5 |
|  | E5CN  (n=1) | 1.05±0.5 | ND | ND | 1.03±0.4 | 87.9 | 20.9 | 110.8 | 127 | 7.6 |

*CN: Control Nitrogen

**ALE: Adaptive laboratory evolution

*** ND: Not detected

**Supplementary Table 2:** Proposed stoichiometries of the glucose and glycerol anaerobic conversion when propionate is oxidized via the methyl malonyl-CoA and the dismutation pathway (*Smithella*) in the case of glucose and glycerol

| **Reaction** | **Stoichiometry** |
| --- | --- |
| **Methyl malonyl-CoA pathway** | |
| Glucose fermentation |  |
| Propionate oxidation |  |
| Butyrate oxidation: |  |
| Aceticlastic methanogenesis |  |
| Hydrogenotrophic methanogenesis |  |
| Glucose (including syntrophic conversions) |  |
| **Dismutation pathway** | |
| Glucose fermentation |  |
| Propionate oxidation |  |
| Butyrate oxidation |  |
| Aceticlastic methanogenesis |  |
| Hydrogenotrophic methanogenesis |  |
| Glucose (via dismutation pathway and including syntrophic conversions) |  |
| **Methyl malonyl-CoA pathway** | |
| Glycerol fermentation |  |
| Propionate oxidation |  |
| Aceticlastic methanogenesis |  |
| Hydrogenotrophic methanogenesis |  |
| Glycerol (including syntrophic conversions) |  |
| **Dismutation pathway** | |
| Glycerol fermentation |  |
| Propionate oxidation |  |
| Butyrate oxidation |  |
| Aceticlastic methanogenesis |  |
| Hydrogenotrophic methanogenesis |  |
| Glycerol (via dismutation pathway and including syntrophic conversions) |  |

**Supplementary Table 3:** Effect of substrate concentration in the water activity of the liquid medium employed in the experiments of anaerobic conversion of glucose and glycerol under elevated pCO_2_

| **Component** | | | **Water activity (a_w_)** | **Volume atm reactor (mL)** | **Volume pressure reactor (mL)** | **Remarks** |
| --- | --- | --- | --- | --- | --- | --- |
| Anaerobic sludge | | | 0.992 | 20 | 14 | (Agoda-Tandjawa et al., 2013)  Solids content in the sludge ≈1.5% w/w |
| Concentrated Macronutrients and micronutrients solution | | | 0.870 | 0.90 | 0.72 | Calculated in PhreeqC based on the composition indicated by Garcia-Rea et. al., (2020) |
| Substrate solution + 100 mM HCO_3_^-^ | | |  | 130 | 106 | Experiments (this work) |
|  | | | **a_w_** | **a_w_ atm. reactor** | **a_w_ pressure reactor** | a_w_ is calculated from an approcimation based on Raoult’s law  (Parkhurst and Appelo, 1999) |
| Glucose Concentration (mM) | 0 | | 1 | 0.998 | 0.997 | Calculated in PhreeqC |
|  | 50 | | 1.00 | 0.994 | 0.993 |  |
|  | 100 | | 0.99 | 0.994 | 0.993 |  |
|  | 1000 | | 0.97 | 0.977 | 0.978 |  |
|  | 2500 | | 0.92 | 0.925 | 0.933 |  |
| Glycerol Concentration (mM) | 0 | | 1 | 0.998 | 0.997 | Calculated in PhreeqC |
|  | 100 | | 0.99 | 0.994 | 0.993 |  |
|  | 200 | | 0.99 | 0.992 | 0.991 |  |
|  | 2000 | | 0.96 | 0.960 | 0.963 |  |
|  | 5000 | | 0.86 | 0.876 | 0.890 |  |
|  | Pr  (mM) | Ac (mM) |  | | |  |
| VFA mixture  (Propionate - Pr + Acetate - Ac) | 0 | 0 | 1 | 0.998 | 0.998 | Calculated in PhreeqC |
|  | 100 | 50 | 1.00 | 0.994 | 0.995 |  |
|  | 200 | 100 | 0.99 | 0.992 | 0.992 |  |
|  | 1000 | 500 | 0.97 | 0.972 | 0.972 |  |
|  | 5000 | 2500 | 0.74 | 0.771 | 0.767 |  |

# References

Agler, M. T., Wrenn, B. A., Zinder, S. H., and Angenent, L. T. (2011). Waste to bioproduct conversion with undefined mixed cultures: the carboxylate platform. *Trends Biotechnol.* 29, 70–78. doi:10.1016/j.tibtech.2010.11.006.

Agoda-Tandjawa, G., Dieudé-Fauvel, E., Girault, R., and Baudez, J. C. (2013). Using water activity measurements to evaluate rheological consistency and structure strength of sludge. *Chem. Eng. J.* 228, 799–805. doi:10.1016/j.cej.2013.05.012.

Ammar, E. M., Jin, Y., Wang, Z., and Yang, S. T. (2014). Metabolic engineering of Propionibacterium freudenreichii: Effect of expressing phosphoenolpyruvate carboxylase on propionic acid production. *Appl. Microbiol. Biotechnol.* 98, 7761–7772. doi:10.1007/s00253-014-5836-y.

Bokulich, N. A., Subramanian, S., Faith, J. J., Gevers, D., Gordon, J. I., Knight, R., Mills, D. A., and Caporaso, J. G. (2013). Quality-filtering vastly improves diversity estimates from Illumina amplicon sequencing. *Nat. Methods* 10, 57–59. doi:10.1038/nmeth.2276.

Caporaso, J. G., Kuczynski, J., Stombaugh, J., Bittinger, K., Bushman, F. D., Costello, E. K., et al. (2010). QIIME allows analysis of high-throughput community sequencing data. *Nat. Methods* 7, 335–336. doi:10.1038/nmeth.f.303.

Edgar, R. C. (2004). MUSCLE: multiple sequence alignment with high accuracy and high throughput. *Nucleic Acids Res.* 32, 1792–1797. doi:10.1093/nar/gkh340.

Edgar, R. C. (2013). UPARSE: highly accurate OTU sequences from microbial amplicon reads. *Nat. Methods* 10, 996–998. doi:10.1038/nmeth.2604.

Edgar, R. C., Haas, B. J., Clemente, J. C., Quince, C., and Knight, R. (2011). UCHIME improves sensitivity and speed of chimera detection. *Bioinformatics* 27, 2194–2200. doi:10.1093/bioinformatics/btr381.

García Rea, V. S., Muñoz Sierra, J. D., Fonseca Aponte, L. M., Cerqueda-Garcia, D., Quchani, K. M., Spanjers, H., and van Lier, J. B. (2020). Enhancing Phenol Conversion Rates in Saline Anaerobic Membrane Bioreactor Using Acetate and Butyrate as Additional Carbon and Energy Sources. *Front. Microbiol.* 11. doi:10.3389/fmicb.2020.604173.

Magoc, T., Salzberg, S. L., Magoč, T., and Salzberg, S. L. (2011). FLASH: fast length adjustment of short reads to improve genome assemblies. *Bioinformatics* 27, 2957–2963. doi:10.1093/bioinformatics/btr507.

Parkhurst, D. L., and Appelo, C. A. J. (1999). USER’S GUIDE TO PHREEQC (VERSION 2). Available at: http://www.xs4all.nl/~appt/index.html.

Quast, C., Pruesse, E., Yilmaz, P., Gerken, J., Schweer, T., Yarza, P., Peplies, J., and Glöckner, F. O. (2012). The SILVA ribosomal RNA gene database project: improved data processing and web-based tools. *Nucleic Acids Res.* 41, D590–D596. doi:10.1093/nar/gks1219.

Wang, Q., Garrity, G. M., Tiedje, J. M., and Cole, J. R. (2007). Naive Bayesian Classifier for Rapid Assignment of rRNA Sequences into the New Bacterial Taxonomy. *Appl. Environ. Microbiol.* 73, 5261–5267. doi:10.1128/AEM.00062-07.

Zhu, C., Nomura, C. T., Perrotta, J. a, Stipanovic, A. J., and Nakas, J. P. (2009). Production and characterization of poly-3-hydroxybutyrate from biodiesel-glycerol by Burkholderia cepacia ATCC 17759. *Biotechnol. Prog.* 26, 424–30. doi:10.1002/btpr.355.
